# Species comparison: human and minipig PBMC reactivity under the influence of immunomodulating compounds *in vitro*

**DOI:** 10.3389/fimmu.2023.1327776

**Published:** 2024-01-09

**Authors:** Clara P. S. Pernold, Emil Lagumdzic, Maria Stadler, Marlies Dolezal, Sven Jäckel, Michael W. Schmitt, Kerstin H. Mair, Armin Saalmüller

**Affiliations:** ^1^ Institute of Immunology, Department of Pathobiology, University of Veterinary Medicine, Vienna, Austria; ^2^ Platform for Bioinformatics and Biostatistics, Department of Biomedical Sciences, University of Veterinary Medicine, Vienna, Austria; ^3^ Chemical and Preclinical Safety, Merck KGaA, Darmstadt, Germany

**Keywords:** minipigs, immunomodulating compounds, comparative study, *in vitro* reactivity, proliferation

## Abstract

Considering the similarities between swine and humans, it is a logical consequence to use swine as a translational model in research and drug development, including non-clinical safety. Here, we compared the reactivity of peripheral blood mononuclear cells (PBMCs) from humans and minipigs under the influence of different compounds *in vitro*. We conducted a flow cytometry-based proliferation assay that focused on the T-cell response to three different stimuli: concanavalin A (ConA), phytohemagglutinin-L (PHA-L), and staphylococcal Enterotoxin B (SEB). Furthermore, four approved immunosuppressive drugs—abatacept, belatacept, rapamycin, and tofacitinib—which are used for the treatment of rheumatoid arthritis or rejection in transplant recipients, were combined with the different stimuli. This allowed us to study the effect of suppressive drugs in comparison with the different stimuli in both species. We examined proliferating T cells (CD3^+^) and investigated the presence of TCR-αβ^+^ and TCR-γδ^+^ T cells. Differences in the response of T cells of the two species under these various conditions were evident. CD4^+^ T cells were more activated within humans, whereas CD8^+^ T cells were generally more abundant in swine. The effectiveness of the used humanized antibodies is most likely related to the conserved structure of CTLA-4 as abatacept induced a much stronger reduction in swine compared with belatacept. The reduction of proliferation of rapamycin and tofacitinib was highly dependent on the used stimuli. We further investigated the effect of the immunosuppressive compounds on antigen-specific restimulation of pigs immunized against porcine circovirus 2 (PCV2). Treatment with all four compounds resulted in a clear reduction of the proliferative response, with rapamycin showing the strongest effect. In conclusion, our findings indicate that the effectiveness of suppressive compounds is highly dependent on the stimuli used and must be carefully selected to ensure accurate results. The results highlight the importance of considering the response of T cells in different species when evaluating the potential of an immunomodulatory drug.

## 1 Introduction

Humans and swine share many similarities, making the swine an important model organism for human disease and even a potential organ source (1–6). As pointed out in the review by Käser, a model organism has to be accessible and affordable, and results need to be translational to the species of interest (7). Here, Göttingen minipigs (GMs), a breed established particularly to address research questions, have proven to be a valuable model in experimental medicine and toxicology (8–15). In addition, the generation of genetically modified animals over the last years offers an even broader usability of the GM model (16–18). A vast advantage over domestic swine is the smaller size, which results in a reduced amount of compound needed in *in-vivo* studies. Although an appreciated research species, information on the GM immune system is still limited. Recently, our group has investigated the postnatal maturation of the immune system of GMs (19). We studied the postnatal development under SPF conditions, as well as under normal housing conditions and after vaccination. These studies showed a change in the composition of leukocyte populations, particularly T-cell subsets, from naive to more experienced phenotypes.

*Ex-vivo* analyses establish an important basis for the use of peripheral blood mononuclear cells (PBMCs) for lymphocyte cultures for the analysis of immunomodulatory compounds. These minipig PBMC cultures allow screening of potential candidates of immunomodulatory compounds and, thus, will lead to a reduction of studies on living animals in the context of the 3Rs, especially when using cryopreserved samples (20). The use of improved reagents, methods, and equipment to define and characterize an immune response in the form of fluorescent reagents for tracking proliferation after stimulation and monoclonal antibodies to characterize the phenotype of reactive cells and the analysis of cytokine production as well as transcription factors are essential. Those can provide a detailed insight into an *in-vitro* immune response which contributes significantly to another component of the 3Rs principle: refinement. To characterize the influence of immunomodulatory substances on porcine PBMC cultures, we used PBMCs of six GMs for *in-vitro* studies and compared the observed effects to human PBMC cultures. We assessed the *in-vitro* stimulation capacity for porcine and human PBMCs using three different well-established T-cell stimuli: the T-cell mitogens concanavalin A (ConA) and phytohemagglutinin-L (PHA) and the superantigen staphylococcal enterotoxin B (SEB) (21–23). Furthermore, we tested the *in-vitro* effect of four immune suppressive drugs used in human medicine for the treatment of rheumatoid arthritis and rejection in transplant recipients (24–26). Abatacept and belatacept inhibit T-cell activation by binding to CD80/86, which interacts with CD28 to provide the so-called “second signal” required for T-cell activation (27–30). Additionally, we used rapamycin which inhibits a signaling pathway responsible for lymphocyte growth, proliferation, and survival by binding to the mechanistic target of rapamycin (mTOR) (31–33). Furthermore, we used the Janus kinase (JAK) inhibitor tofacitinib. JAKs, cytoplasmic tyrosine kinases, are known to play important roles in many cellular processes such as the modulation of the CD80/CD86 expression on LPS-stimulated DCs. Therefore, the chosen compounds will target the effects of the selected stimuli (for example abatacept/belatacept and SEB, rapamycin and PHA, tofacitinib and ConA) (24, 34–39).

We analyzed a total of 15 different compound groups to gain insight into the effect of the stimuli on different T-cell subpopulations in the two species and whether the effect of oligo- and polyclonal stimuli in combination with the different suppressors plays an important role. The obtained results will establish and improve baseline information on the GM immune system and expand the toolbox for the use of GMs as a model organism for compound testing important for humans. In addition, possible interfering effects of the respective stimuli and immunosuppressive drugs need to be considered. To investigate the inhibitory potential of drugs targeting a specific part of T-cell activation like abatacept or belatacept, we combined them with T-cell mitogens to gain information on possible interactions. We propose that the use of different combinations of stimuli/suppressors provides valuable insights into the *in-vitro* mode of action of these agents and thus their efficacy. Our data illustrate that stimulation *in vitro* must be carefully evaluated to provide translatable results between both species. Indecisive effects of the tested suppressors can be due to the stimulatory compound chosen rather than the suppressor itself. Therefore, the choice of the stimulatory compound must be carefully evaluated, and its usability in the assay must be confirmed.

In our *in-vitro* experiments, we investigated the effect of the immunomodulatory compounds by proliferation assays using multicolor flow cytometry (FCM) to determine T-cell ratios within proliferating lymphocytes. At first, we differentiated between all T cells (all CD3^+^ lymphocytes), T-cell receptor (TCR)-αβ^+^ T cells, and TCR-γδ^+^ T cells, as swine is a species with a high abundance of TCR γδ T cells compared with human (40–43). Furthermore, we analyzed the presence of CD4^+^ and CD8^+^ T cells within the proliferating TCR-αβ^+^ T-cell population.

## 2 Material and methods

### 2.1 Samples and cell isolation

#### 2.1.1 Animals

Six adult Ellegaard Göttingen minipigs (GMs, three males and three females) were used in this study. The breed of GMs has been established in the 1960s in Göttingen, Germany. The background of these pigs is Minnesota Minipigs from Hormel Institute, Austin, TX, USA; Vietnamese potbellied pigs from Wilhelma Zoo, Stuttgart, Germany, and Friedrichsfelde Zoo, Berlin, Germany; and the German Landrace (44). During their time at our facility, all GMs were housed together under conventional conditions. The male pigs were castrated, and all animals were vaccinated against *Mycoplasma hyopneumoniae* (Ingelvac MycoFLEX^®^, Boehringer Ingelheim Vetmedica GmbH, Ingelheim, Germany), porcine circovirus 2 (PCV2) (Ingelvac CircoFLEX^®^, Boehringer Ingelheim Vetmedica GmbH), *Actinobacillus pleuropneumoniae* (APP) (COGLAPIX^®^, CEVA Tiergesundheit GmbH, Dessau, Germany), and a stock-specific vaccine against *Glaesserella parasuis* (serotype 4, BS-Immun GmbH, Vienna, Austria). The animal study was approved by the Advisory Committee for Animal Experiments of the University of Veterinary Medicine Vienna (§12 Animal Experiments Act - TVG) and the Austrian Federal Ministry of Education, Science and Research (reference BMBWF-68.205/0198-V/3b/2019). Blood was collected in heparin tubes (Primavette^®^ V Li-Heparin 10 mL; Kabe Labortechnik GmbH, Nümbrecht-Elsenroth, Germany), and PBMCs were isolated by density gradient centrifugation (Pancoll human, density 1.077 g/mL, PAN-Biotech, Aidenbach, Germany) 30 min at 920×g. Absolute cell counts were determined with a Sysmex XP300 (Sysmex Austria GmbH, Vienna, Austria). Isolated PBMCs were stored at −150°C in a freezing medium containing 50% (v/v) RPMI 1640 with stable glutamine (PAN-Biotech) supplemented with 100 IU/mL of penicillin and 0.1 mg/mL of streptomycin (PAN-Biotech), 40% (v/v) fetal calf serum (FCS, Gibco™, Thermo Fisher Scientific, Vienna, Austria), and 10% (v/v) DMSO (Sigma-Aldrich, Burlington, MA, USA).

#### 2.1.2 Human samples

Na-heparin blood samples of healthy adult human donors (three women and three males) were obtained from Red Cross Austria. Usage is regulated through “Richtlinie zur Verwendung und Weitergabe von Materialien menschlichen Ursprungs für andere Zwecke als für die Transfusion oder Transplantation beziehungsweise zur Herstellung von Humanarzneimitteln” and internally through the Ethics Commission of Red Cross Austria. PBMCs were isolated as described above in Section 2.1.1, as well as the determination of cell counts and freezing.

### 2.2 Stimulation assays and staining

#### 2.2.1 Staining of PBMCs with CellTrace™ Violet stain

The staining protocol was described previously (19). In short, PBMCs were defrosted in D-PBS (PAN-Biotech) and filtered, and 2 × 10^7^ cells/mL were stained with 1 mL of a 5-µM CellTrace™ Violet solution (CTV, Thermo Fisher Scientific) followed by instant vortexing. Incubation lasted for 10 min in a 37°C water bath with repeated vortexing, followed by adding 2 mL of FCS per 2 mL of CTV/cell suspension and 15 min incubation in the dark. After that, cells were washed three times with cell culture medium (RPMI, 10% FCS, 100 IU/mL penicillin, and 0.1 mg/mL streptomycin).

#### 2.2.2 Stimulation and surface staining

Cells/well (2 × 10^5^) were plated into 96-well round-bottom plates (Greiner Bio-One, Kremsmünster, Austria) and stimulated either with 3 μg/mL of ConA (Amersham Bioscience AB, Uppsala, Sweden), 5 μg/mL of PHA (Roche, Basel, Switzerland), or 500 ng/mL of SEB (Sigma Aldrich). For the antigen-specific restimulation, labeled porcine PBMCs from PCV2-vaccinated animals were stimulated with recombinant PCV2-ORF2 protein or GP64 as a baculovirus-expressed control protein (both 4 μg/mL, kindly provided by Boehringer-Ingelheim Vetmedica GmbH) and cultured for 4 days (37°C, 5% CO_2_). In addition, all stimuli were combined with one of the following immune modulators: abatacept (2.5 μg/mL, Bristol-Myers Squibb, New York, USA), belatacept (NULOJIX^®^, 5 μg/mL, Bristol-Myers Squibb), rapamycin (10 ng/mL, Sigma Aldrich), or tofacitinib (500 nM/mL, Sigma Aldrich) and incubated for 5 days (37°C, 5% CO_2_). PBMCs cultivated in medium alone served as negative controls. On day 5 of cultivation, microcultures were harvested and 12 wells of the same stimulation group were pooled and washed twice with D-PBS (PAN-Biotech) supplemented with 3% (v/v) FCS (Gibco™) before staining with monoclonal antibodies presented in **Table 1**. All washing steps of the staining procedure were performed at 470×g, at 4°C for 4 min with 200 µL of the appropriate wash buffer [D-PBS with 3% (v/v) FCS]. Staining lasted for 20 min at 4°C in the dark. In addition, dead cells were stained using fixable viability dye (FVD) according to the manufacturer’s instructions. The viability of the cells was ≥90% after defrosting and in both species, but GMs showed higher viability after *in-vitro* culture.

**Table 1:** Antibodies used for FCM analyses.

Antigen	Clone	Isotype	Fluorochrome	Source of primary antibody	Labeling strategy
human PBMCs					
CD3	SP34-2	Mouse IgG1	Alexa 488	BD Biosciences	Directly conjugated
CD4	L200	Mouse IgG1	APC	BD Biosciences	Directly conjugated
CD8α	RPA-T8 (RUO)	Mouse IgG1	PE-Cy7	BD Biosciences	Directly conjugated
TCR-γδ	B1 (RUO)	Mouse IgG1	BV606	BD Biosciences	Directly conjugated
FVD			eFluor780	Thermo Fisher Scientific	
GM PBMCs					
CD3	BB23-8E6-8C8	Mouse IgG2a	PE-Cy7	BD Biosciences	Directly conjugated
CD4	74-12-4	Mouse IgG2b	PerCP-Cy5.5	BD Biosciences	Directly conjugated
CD8β	PPT23	Mouse IgG1	Alexa 488	In-house	Directly conjugated
TCR-γδ	PPT16	Mouse IgG2b	Alexa 647	In-house	Directly conjugated
FVD			eFluor780	Thermo Fisher Scientific	

### 2.3 Analyses

#### 2.3.1 Data analyses

Samples were analyzed using a CytoFLEX LX (Beckman Coulter, Brea, CA, USA) equipped with six lasers (U-V-B-Y-R-I). At least 200,000 lymphocytes were analyzed per sample. For compensation, single stains were used to set up a compensation library. FCS files were analyzed with FlowJo™ software version 10.8. (Becton Dickinson, Franklin Lakes, NJ, USA). Percentages in the graphs refer to living lymphocytes.

#### 2.3.2 Statistical analyses

All statistical data analyses were performed in R v4.0.5 R Core Team (45). Data were prepared for analysis using functions from packages dplyr v1.0.7 (46) and tidyverse v1.3.1 (47).

We analyzed arcsine square-root-transformed frequencies of CD3^+^, CD4^+^, CD8^+^, and TCR-αβ^+^ T cells via univariate linear mixed-effects models applying function *lmer* in package *lmerTest* v3.1-3 (48) fitting a fixed categorical effect of species with the two-factor levels—human and minipig—and a fixed categorical treatment effect for three stimulators and the combinations of each stimulator with four repressors each, resulting in 15 factor levels, namely, ConA, ConA_Abatacept_, ConA_Belatacept_, ConA_Rapamycin_, ConA_Tofacitinib_, PHA, PHA_Abatacept_, PHA_Belatacept_, PHA_Rapamycin_, PHA_Tofacitinib_, SEB, SEB_Abatacept_, SEB_Belatacept_, SEB_Rapamycin_, and SEB_Tofacitinib_, respectively. Frequencies measured in the medium control were fitted as covariates.

The key in our models is a fitted interaction between the fixed categorical effects of species and treatment. A random intercept for individual (total of 12 factor levels with 6 samples per species) was added to account for the covariance structure in our data, i.e., cells from the same specimen were treated with the different stimulator–repressor drug combinations. We set option *REML* to false to get maximum likelihood estimates of the fixed effects part of our model.

Normal distribution of random effects and residuals and variance homoscedasticity of residuals were verified via diagnostic plots.

We then calculated estimated marginal means for each species–treatment combination using function *emmeans* in package *emmeans 1.7.5* (49) and requested hypothesis testing for all pairwise contrasts using option pairwise. Default multiple testing correction for these pairwise contrasts was turned off (option adjust=“none”). We then filtered for comparisons to the stimulator only level within each group of stimulators. Multiple testing load was therefore 24 tests within each cell type: four contrast of stimulator–repressor combination to the reference level of stimulator alone, times three stimulators, times two species. We performed a false discovery rate (FDR) multiple testing correction (50) within cell type and declare significance at 10% FDR. Please note that in this particular study, choosing a rather high alpha significance cutoff is actually the more conservative approach.

The results of the models are visualized via bar plots of estimated marginal means back-transformed to frequencies using functions make.tran and linkfun on the transformed response within the function call of lmer. Plots were created using functions from packages RColorBrewer v1.1-2 (51), ggplot2 v3.3.5 (52), and ggpubr v0.4.0 (53), in which the fitted model is shown as the height of the bar plot and also black dots and whiskers represent upper and lower 95% confidence intervals of estimated marginal means. Raw data are added to the figures shown as crosses (+). *P*-value brackets display contrasts significant at 10% FD: ****p ≤ 0.001, ***p ≤ 0.01, **p ≤ 0.05, *p ≤ 0.1. Figures were exported as scalable vector graphics using the package svglite v2.0.0 (54).

We refrained from hypothesis testing for TCR-γδ T cells, as they represent a minor population in humans without any response to the three stimuli, and therefore, residuals for this cell type did not meet assumptions for linear mixed-effects models. For PCV2 restimulation assays, no statistics were performed as only three animals were analyzed.

## 3 Results

Multicolor FCM was employed to gain information on the stimulatory capacity of the T-cell mitogens ConA and PHA and the bacterial superantigen SEB. To analyze common reactivities and potential differences between human and GM PBMCs, the proliferation was quantified, and the phenotype of the responding T cells was assessed. To determine the reacting phenotypes, PBMCs stained with CTV™ were stained with mAb against CD3, CD4, CD8, and TCR-γδ after 5 days of cultivation.

**Figure 1:**
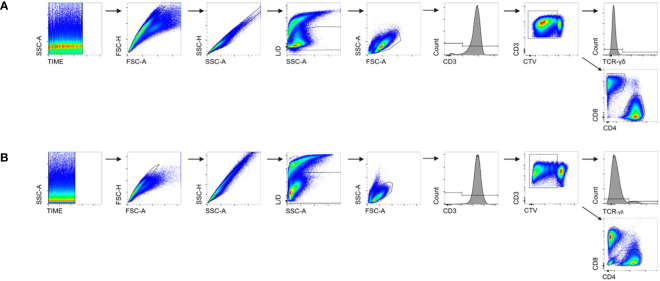
Gating strategy for FCM analyses. **(A)** The gating strategy for human samples; **(B)** the gating strategy for Göttingen minipigs (GMs). A time gate was used to ensure consistent flow rates. To exclude any potential doublets, two doublet discriminations were carried out: FSC-A vs. FSC-H and SSC-A vs. SSC-H. Fixable viability dye was used for live/dead discrimination (L/D) to separate living cells from dead cells and then a lymphocyte gate including blast cells was set. CD3 was used to detect all T cells, and within those, a gate was applied on CellTrace™ (CTV) dim/negative cells to exclude CTV^high+^ resting cells from the analyses. Within the proliferating T cells, TCR-γδ^+^, CD4^+^, and CD8^+^ T cells were analyzed.

The gating strategy and the respective analyses are summarized in **Figure 1**. To ensure consistent flow rates, time vs. SSC-A was set as the first quality criteria, and doublet discrimination was then performed using FSC-A vs. FSC-H together with SSC-H vs. SSC-A. Exclusion of dead cells was achieved by live/dead (L/D) discrimination. Living cells were analyzed as CD3^+^ T cells, and a specific gate was set on proliferating cells showing dilution of CTV™. This fraction was further analyzed for the expression of CD4, CD8, and TCR-γδ. The percentages of the respective fractions were then calculated using FlowJo™ software and are presented in **Table 2**.

**Table 2:** Percentage of proliferating T cells after stimulation with ConA, PHA, and SEB.

	T cells	TCR-αβ T cells	CD4^+^ T cells	CD8^+^ T cells	TCR-γδ T cells
Human					
Medium	4.89 ± 3.58	4.80 ± 3.63	4.35 ± 3.42	0.29 ± 0.25	0.10 ± 0.07
ConA	43.90 ± 16.14	43.52 ± 16.01	30.98 ± 10.59	8.88 ± 7.28	0.39 ± 0.13
PHA	91.08 ± 5.30	90.80 ± 5.26	49.05 ± 14.52	38.77 ± 16.87	0.29 ± 0.12
SEB	80.62 ± 6.14	80.48 ± 6.13	67.45 ± 8.32	10.76 ± 9.06	0.13 ± 0.04
GM					
Medium	9.82 ± 3.25	3.09 ± 1.10	1.20 ± 0.55	1.06 ± 0.52	6.72 ± 3.64
ConA	69.37 ± 6.51	49.72 ± 10.56	12.17 ± 3.32	28.83 ± 6.77	19.65 ± 7.50
PHA	90.32 ± 2.39	85.03 ± 3.65	31.67 ± 6.53	44.27 ± 4.39	5.29 ± 1.96
SEB	48.95 ± 6.70	47.60 ± 6.80	19.48 ± 5.68	22.05 ± 3.90	1.33 ± 0.42

Mean values as well as standard deviations are indicated for all subsets investigated (n = 6).

### 3.1 Stimulation with ConA, PHA, and SEB

Stimulation with the T-cell mitogens ConA and PHA as well as the bacterial superantigen SEB is presented in **Table 2**, which summarizes the mean and standard deviation values of proliferating T cells after the different stimulations and spontaneous proliferation of the control group (medium). The table shows data obtained from FlowJo™ analyses prior to the statistical modeling, thus resulting in percentages after measurement and not yet including medium as a covariant. ConA stimulation resulted in a higher activation of total T cells in PBMCs of GMs (69.37% ± 6.51%) compared with human PBMCs (43.90 ± 16.14). However, the difference between the TCR-αβ T-cell values of the two species was less prominent: human TCR-αβ T cells showed frequencies of 43.52% ± 16.01% of proliferating cells and GMs’ TCR-αβ T cells showed 49.72% ± 10.56%. This difference compared with total T cells could be explained by the proliferation of the TCR-γδ T-cell subpopulation within GMs (19.65% ± 7.50%), whereas proliferating human CD3^+^ cells contained only a minor fraction of TCR-γδ T cells with 0.39% ± 0.13% of proliferating cells. Another difference was obvious within the CD4^+^ and CD8^+^ T-cell subpopulations. Human CD4^+^ T cells showed a higher proliferation (30.98% ± 10.59%) compared with GMs with 12.17% ± 3.32%. Regarding CD8^+^ T cells, the percentages of proliferating cells were reversed between the two species compared with CD4^+^ T cells. In human PBMCs, only 8.88% ± 7.28% proliferating CD8^+^ T cells were detected, whereas GMs showed a clear domination of CD8^+^ proliferating T cells with 28.83% ± 6.77%.

After stimulation with PHA, differences were less prominent compared with ConA. Both species showed a very high reactivity of T cells with 91.08% ± 5.30% for human samples, compared with 90.32% ± 2.39% proliferating porcine T cells. Accordingly, proliferation results of TCR-αβ T cells in humans (90.80% ± 5.26%) were comparable with those in GMs (85.03% ± 3.65%). Comparable to ConA but less prominent, PHA stimulation resulted in a slightly better proliferation of human CD4^+^ cells (49.05% ± 14.52%) compared with CD8^+^ T cells (38.77% ± 16.87%), while the results were opposite in samples from GMs (31.67% ± 6.53% for CD4^+^ T cells and 44.27% ± 4.39% for CD8^+^ T cells). Only a minor increased proliferative response was observed after PHA stimulation within porcine TCR-γδ T cells (5.29% ± 6.96%), while hardly any was observed within this subpopulation in human samples (0.29% ± 0.12%).

SEB stimulation showed a much higher effect on human T cells compared with GMs. This was not only obvious within total T cells (human: 80.62% ± 6.14%, GM: 48.95% ± 6.70%), but also within TCR-αβ T cells (human: 80.48% ± 6.13%, GM: 47.60% ± 6.80%). Within TCR-αβ T cells, especially human CD4^+^ T cells showed a higher proliferative capacity after SEB stimulation (67.45% ± 8.32%), compared with porcine CD4^+^ T cells (19.48% ± 5.68%). In contrast, the results on CD8^+^ T cells showed opposite results (human: 10.78% ± 6.13%, GM: 22.05% ± 3.90%). Of note, TCR-γδ T cells of both species did not show any obvious proliferation after SEB stimulation (human: 0.13% ± 0.04%, GM: 1.33% ± 0.42%).

Studies on the stimulation capacities of the mitogens ConA and PHA together with the bacterial superantigen SEB enable detailed analyses for the further testing of the preselected immunomodulatory compounds: abatacept (A), belatacept (B), rapamycin (R), and tofacitinib (T). As within humans, hardly any proliferation was observed within TCR-γδ T cells. Except for ConA stimulation, the same accounted for porcine samples. Therefore, we further focused on total T cells and TCR-αβ T cells only.

### 3.2 Suppressive effects of immunomodulatory compounds on T-cell proliferation after ConA, PHA, and SEB stimulation

#### 3.2.1 Total T cells

**Figure 2:**
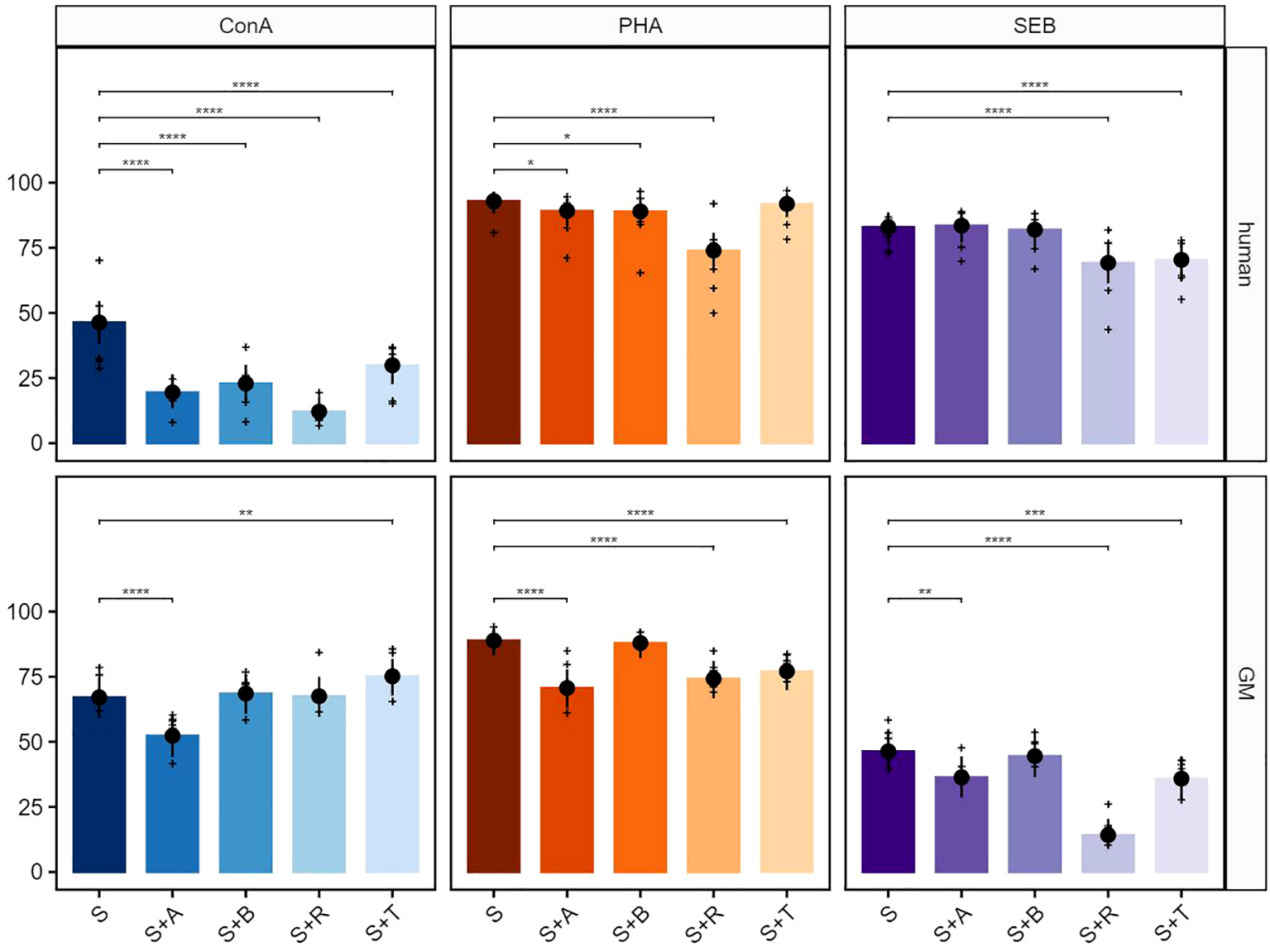
Suppressive effects on T cells after stimulation with concanavalin A (ConA), phytohemagglutinin-L (PHA), and staphylococcal enterotoxin B (SEB). The percentages of proliferating CD3^+^ cells for human (upper row) and GMs (bottom row) are presented after 5 days of *in-vitro* cultivation in combination with four different immunomodulating compounds. S indicates the percentage of the stimulated controls without compound treatment. The compounds are presented by their abbreviations: abatacept (A), belatacept (B), rapamycin (R), and tofacitinib (T). Data are based on six different donors for each species. The height of the bar and black dots correspond to back-transformed estimated marginal means, and whiskers represent the upper and lower 95% confidence intervals of the estimated marginal means. Raw data are shown as (+). *P*-value brackets display contrasts significant at 10% FDR: *p ≤ 0.1, **p ≤ 0.05, ***p ≤ 0.01, ****p ≤ 0.001.

The test compounds abatacept (A), belatacept (B), rapamycin (R), and tofacitinib (T) on ConA-stimulated PBMCs in humans led to a significant reduction of the proliferative response in total T cells (**Figure 2**, upper row, left graph). In GMs, abatacept treatment led to a clear inhibitory effect on the proliferation of T cells, while tofacitinib resulted in an increase in the percentage of proliferating cells. Belatacept and rapamycin showed no statistically relevant inhibitory or stimulating activity (**Figure 2**, bottom row, left graph). As summarized already in **Table 2**, both human- and GM-derived T cells showed a strong reactivity after stimulation with PHA without any compound treatment (**Figure 2**, middle graphs). The proliferation was significantly reduced after treatment with rapamycin in cultures with PBMCs of both species. The same accounted for abatacept although to a lesser extent in human samples. While belatacept showed a slight reduction in humans and not GMs, the biggest difference was observed after tofacitinib treatment. Here, a highly significant reducing effect on porcine cells only was observed. Although SEB stimulation was stronger in human T cells compared with GMs, similar inhibitory effects on proliferation were observed after rapamycin or tofacitinib treatment (**Figure 2**, right graphs). Again, abatacept showed a more prominent effect on porcine T cells. Belatacept did not show any effect in both species.

#### 3.2.2 TCR-αβ T cells

**Figure 3:**
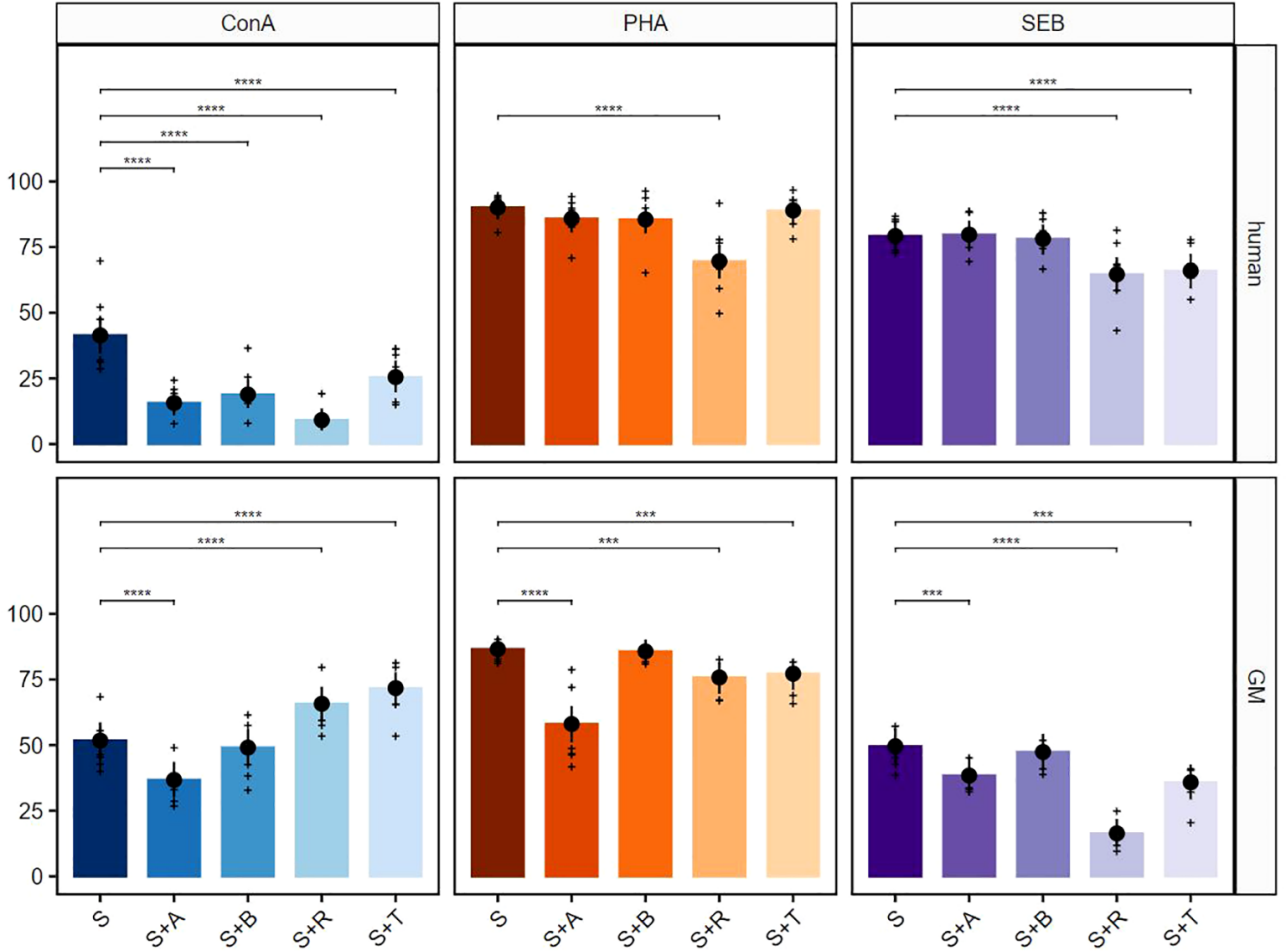
Suppressive effects on TCR-αβ T cells after stimulation with ConA, PHA, and SEB. The percentages of proliferating CD3^+^TCR-γδ^−^ T cells for human (upper row) and GMs (bottom row) are presented after 5 days of *in-vitro* cultivation in combination with four different immunomodulating compounds. S indicates the percentage of the stimulated controls without compound treatment. The compounds are presented by their abbreviations: abatacept (A), belatacept (B), rapamycin (R), and tofacitinib (T). Data are based on six different donors for each species. The height of the bar and black dots correspond to back-transformed estimated marginal means, and whiskers represent the upper and lower 95% confidence intervals of the estimated marginal means. Raw data are shown as (+). *P*-value brackets display contrasts significant at 10% FDR: ***p ≤ 0.01, ****p ≤ 0.001.

In the next step, we performed selective analyses of the response of TCR-αβ T cells (CD3^+^TCR-γδ^−^). After stimulation with ConA, all four suppressors in cultures with human PBMCs generated a strong significant reduction of the proliferative response (**Figure 3**, upper row, left graph). The same accounted for samples derived from GMs for abatacept. Similar to the data derived from total T cells, tofacitinib in combination with ConA had a promoting effect on proliferation. In contrast to total T cells, also this promoting effect was observed in the ConA + rapamycin combination (**Figure 3**, bottom row, left graph). Proliferation of TCR-αβ T cells could be significantly blocked with rapamycin in both species after PHA stimulation. In contrast to human samples, tofacitinib and especially abatacept showed a clear reduction in proliferating TCR-αβ T cells from GMs (**Figure 3**, middle graphs). Rapamycin and tofacitinib treatment showed a significant reduction in the proliferative response after SEB stimulation in both species. In addition, this reduction was observed after abatacept treatment for TCR-αβ T cells from GMs (**Figure 3**, right graphs).

#### 3.2.3 CD4^+^ T cells

**Figure 4:**
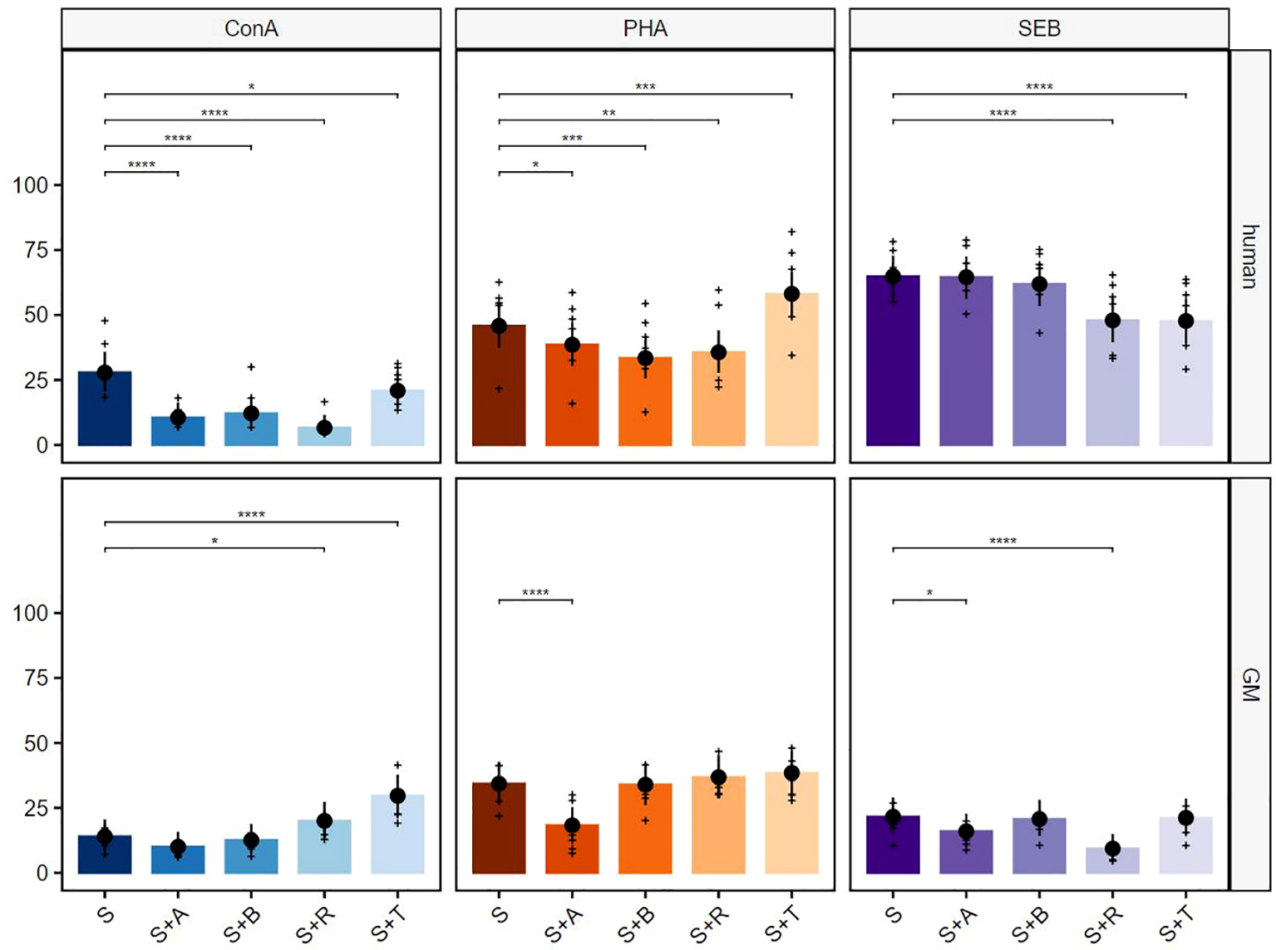
Suppressive effects on CD4^+^ T cells after stimulation with ConA, PHA, and SEB. The percentages of proliferating CD3^+^TCR-γδ^−^CD4^+^ T cells for human (upper row) and GMs (bottom row) are presented after 5 days of *in-vitro* cultivation in combination with four different immunomodulating compounds. S indicates the percentage of the stimulated controls without compound treatment. The compounds are presented by their abbreviations: abatacept (A), belatacept (B), rapamycin (R), and tofacitinib (T). Data are based on six different donors for each species. The height of the bar and black dots correspond to back-transformed estimated marginal means, and whiskers represent the upper and lower 95% confidence intervals of the estimated marginal means. Raw data are shown as (+). *P*-value brackets display contrasts significant at 10% FDR: *p ≤ 0.1, **p ≤ 0.05, ***p ≤ 0.01, ****p ≤ 0.001.

**Figure 5:**
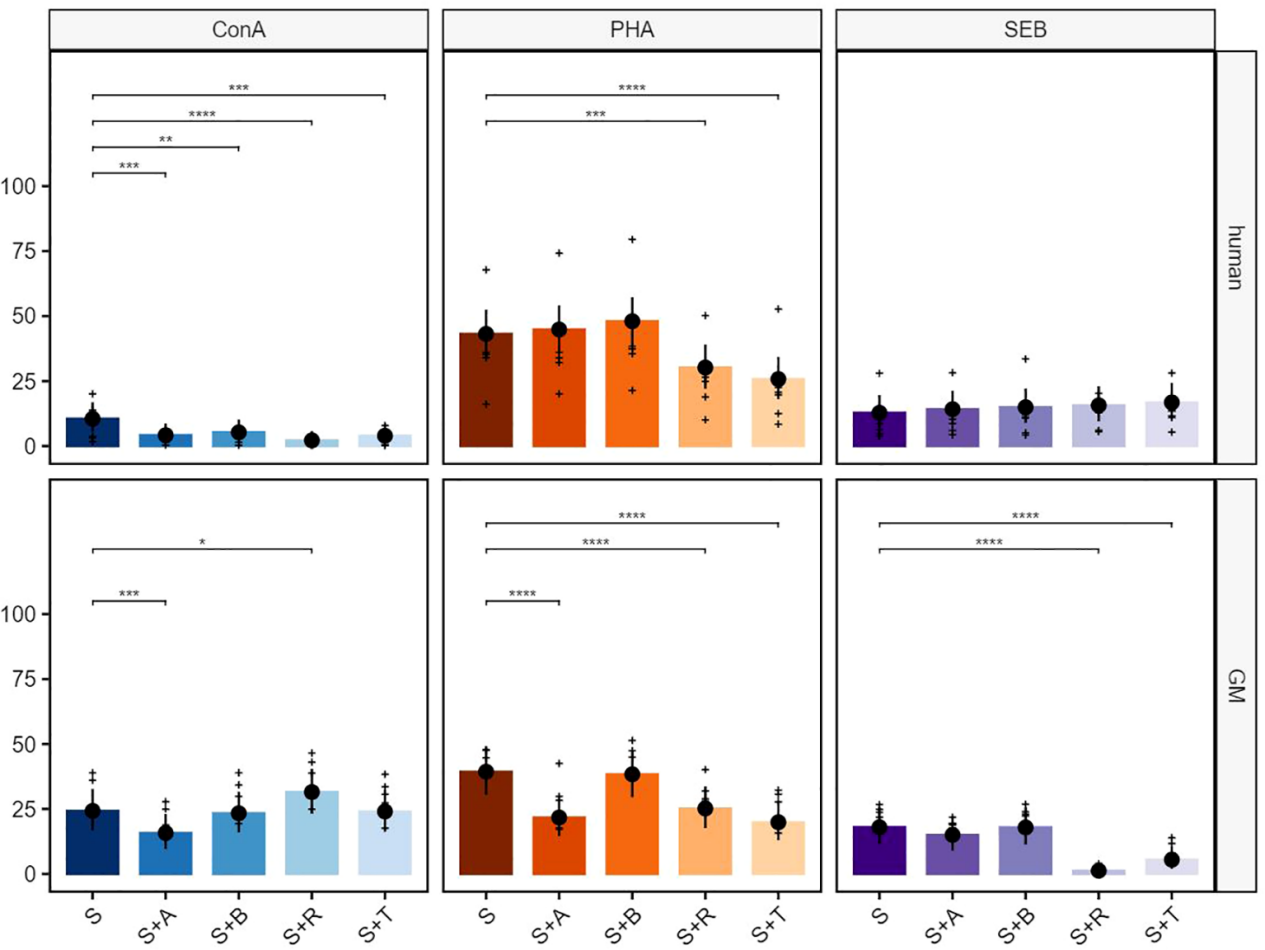
Suppressive effects on CD8^+^ T cells after stimulation with ConA, PHA, and SEB. The percentages of proliferating CD3^+^TCR-γδ^−^CD8^+^ T cells for human (upper row) and GMs (bottom row) are presented after 5 days of *in-vitro* cultivation in combination with four different immunomodulating compounds. S indicates the percentage of the stimulated controls without compound treatment. The compounds are presented by their abbreviations: abatacept (A), belatacept (B), rapamycin (R), and tofacitinib (T). Data are based on six different donors for each species. The height of the bar and black dots correspond to back-transformed estimated marginal means, and whiskers represent the upper and lower 95% confidence intervals of the estimated marginal means. Raw data are shown as (+). *P*-value brackets display contrasts significant at 10% FDR: *p ≤ 0.1, **p ≤ 0.05, ***p ≤ 0.01, ****p ≤ 0.001.

For a more detailed characterization of responding cell populations, proliferative CD3**^+^** T cells were further analyzed regarding their CD4 (**Figure 4**) and CD8 (**Figure 5**) expression. The analyses of proliferating CD4^+^ T cells after ConA stimulation in combination with abatacept, belatacept, rapamycin and, to a lesser extent, with tofacitinib resulted in a comparable picture regarding the inhibitory activity within human proliferating TCR-αβ T cells (**Figure 4**, upper row, left graph). For porcine CD4**^+^** T cells, no significant immunosuppressive effects of abatacept or belatacept on the proliferative response were detected after ConA stimulation. Interestingly, after treatment with rapamycin or tofacitinib, a significant increase in the percentage of proliferating CD4**^+^** T cells occurred (**Figure 4**, bottom row, left graph). After PHA stimulation, abatacept, belatacept, and rapamycin led to a decrease of proliferating cells (**Figure 4**, upper row middle graph). In contrast, a promoting effect was detected after treatment with tofacitinib. Within porcine CD4**^+^** T cells, only abatacept showed a significant reduction of the proliferative response (**Figure 4**, bottom row, middle graph). After SEB stimulation, an inhibitory effect within proliferating human CD4**^+^** cells was only detectable in combination with rapamycin or tofacitinib (**Figure 4**, upper row, right graph). Within GMs, only rapamycin and to a lesser extent abatacept showed a significant reduction of proliferating CD4**^+^** T cells (**Figure 4**, bottom row, right graph).

#### 3.2.4 CD8^+^ T cells

In the next step, CD8^+^ T cells were analyzed in detail (**Figure 5**). All four tested compounds showed a significant reduction of human proliferating CD8^+^ T cells after ConA stimulation (**Figure 5**, upper row, left graph). This effect could not be shown for CD8^+^ porcine T cells, as only abatacept induced a significant reduction. Rapamycin, on the other hand, showed an increasing effect on CD8^+^ T cells (**Figure 5**, bottom row, left graph). Both the PHA-stimulated human and porcine PBMCs showed significant inhibition of the proliferation of the CD8^+^ T cells after rapamycin as well as tofacitinib treatment. Furthermore, an inhibitory effect of the abatacept treatment was visible within the porcine samples (**Figure 5**, middle graphs). CD8^+^ human T cells showed weak reactivity after stimulation with SEB, and hardly any effect of the immunomodulatory compounds was visible (**Figure 5**, upper row, right graph). In contrast, significant inhibitory effects on the percentage of porcine CD8^+^ proliferating cells were shown for rapamycin and tofacitinib after stimulation with SEB (**Figure 5**, bottom row, right graph).

#### 3.2.5 Impact of immunomodulatory compounds on antigen-specific restimulation in GMs

**Figure 6:**
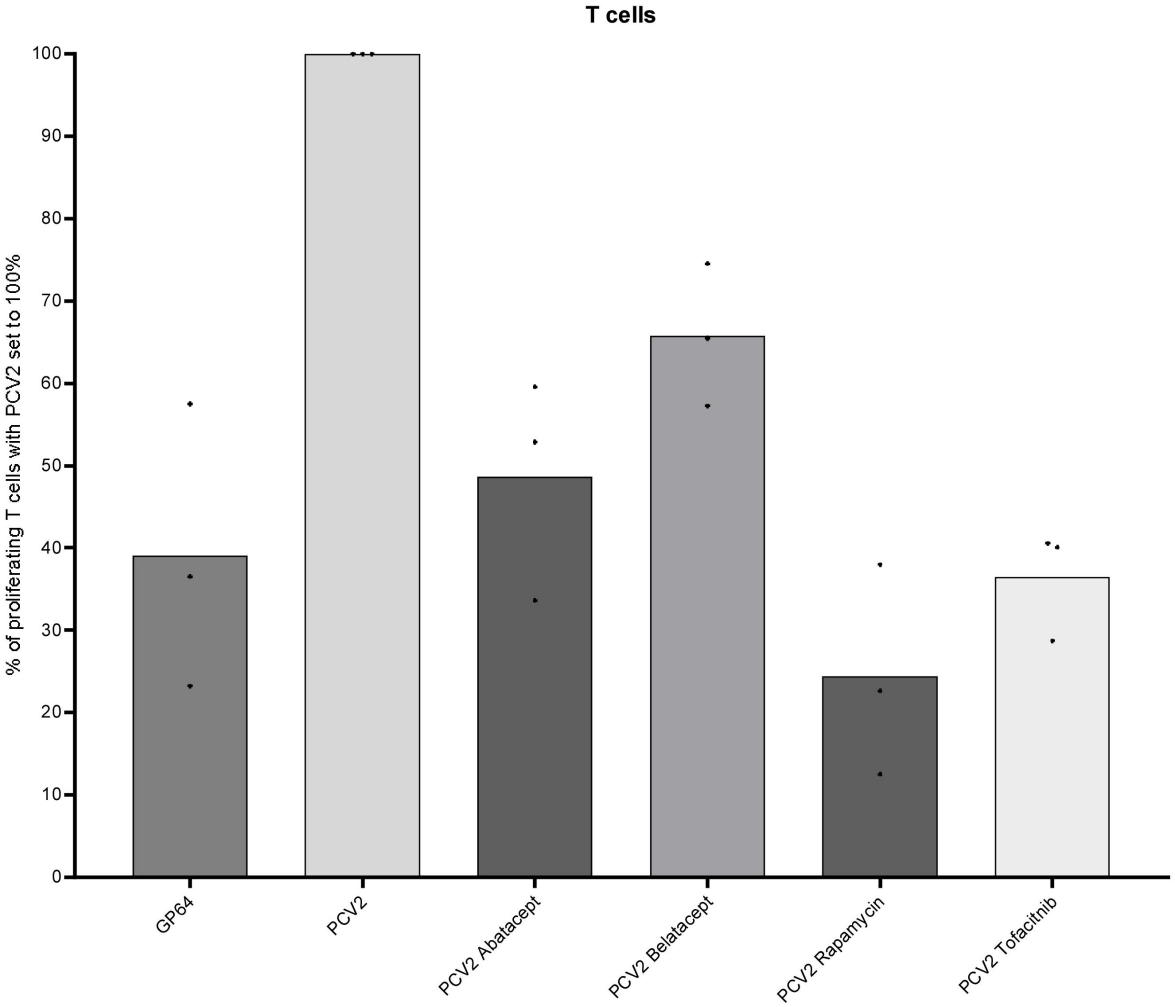
Suppressive effects on GMs’ T cells after PCV2 antigen-specific restimulation. Proliferative response of PCV2-restimulated T cells (CD3^+^) of three MGs is shown after 4 days of *in-vitro* cultivation and in combination with the four different immunomodulating compounds—abatacept, belatacept, rapamycin, and tofacitinib. In addition, baculovirus-expressed GP64 was used as a negative control.

As we have shown in Section 3.2, drug efficacy is highly dependent on stimuli; hence, we investigated the effect of antigen-specific restimulation in the minipig model. As it was not possible to test on the same antigens as in humans, we opted for PCV2 restimulation as GMs were vaccinated in week 29 (19). They received further booster vaccinations in week 33 and 10 months later. We used PBMCs from three pigs 16 months after the last PCV2 boost and restimulated them as described above. After 4 days, we harvested the cells and analyzed the proliferating T cells. Restimulation with baculovirus-expressed recombinant PCV2-ORF2 led to a distinct proliferation of T cells (8.1% ± 1.1%; data not shown). PCV2-responding T cells were set to 100% for the following analyses. A group restimulated with an empty baculovirus vector (GP64) was included as a control group and showed 60% reduced proliferative response compared with the PCV2 group. Likewise, all four immunomodulatory compounds led to a high inhibition of proliferation (**Figure 6**). The lowest reduction was observed after belatacept treatment (35%), followed by abatacept (50%) and tofacitinib (64%). Treatment of cells with rapamycin even showed a reduction of 75% of proliferating T cells.

## 4 Discussion

It has been widely discussed how well animal models are suited for studying human diseases and the challenges that researchers have to address (55–59). Although many attempts have been made to replace animal models, it is not yet possible to completely eliminate them in the development of new drug candidates (60, 61). To fulfill the concept of the 3Rs, it is essential to optimize animal models (20). To increase knowledge on *in-vitro* activation of cell subsets, we compared the responsiveness of human and minipig PBMCs *in vitro* after stimulation with two T-cell mitogens (ConA and PHA) and one bacterial superantigen (SEB) and studied the influence of immunomodulatory compounds on their proliferative capacity. T cells of both species can be stimulated very well with ConA, PHA, or SEB. Porcine T cells responded better to ConA than human samples. PHA stimulation on the other hand showed a more comparable response between the species, while SEB stimulation led to a higher percentage of proliferating cells in humans compared with the pig. When having a closer look at the distinct T-cell subset, some differences were observed between species. Porcine CD4^+^ T cells responded less to ConA stimulation than human CD4^+^ T cells, while human CD8^+^ T cells responded less to SEB stimulation than porcine CD8^+^ T cells. In an earlier publication, we characterized the immune system of EGMs in detail and found fewer CD4 T cells in our adult pigs compared with published human data (19). This difference could be an influential factor as we do see more CD4 T cells within all stimuli in human samples compared with our pigs. For SEB, we speculate that the different reactivity of human and minipig CD8 T cells can be explained by the binding to different alleles of MHC class II molecules and variable regions of the β-chains of T-cell receptors (62, 63). This might promote a better response of minipig cells, thus indicating that different responses within the two TCR-αβ T-cell subpopulations exist depending on the stimuli and proposing PHA as the best stimulation to compare proliferative response between the two species. Human TCR-γδ T cells did not respond at all to any of the three stimuli. Porcine TCR-γδ T cells only showed increased proliferative response over the medium control after ConA stimulation. This is probably due to the fact that the minipig belongs to the so-called “γδ high” species compared with humans as “γδ low” species (64). As a direct comparison of the compounds’ influences was not possible due to the lack of responsiveness of human PBMCs after ConA, PHA, and SEB stimulation, we excluded this subset for further data analyses and focused on TCR-αβ T cells. Although responses on TCR-γδ T cells could not be compared in this study, minipigs might provide an opportunity to study the effects of other stimuli in combination with compounds on TCR-γδ T cells which can be beneficial for the role of TCR-γδ T cells in human diseases (65–67). To study the potential effects on human TCR-γδ T cells, enrichment by cell sorting could be conducted.

**Figure 7:**
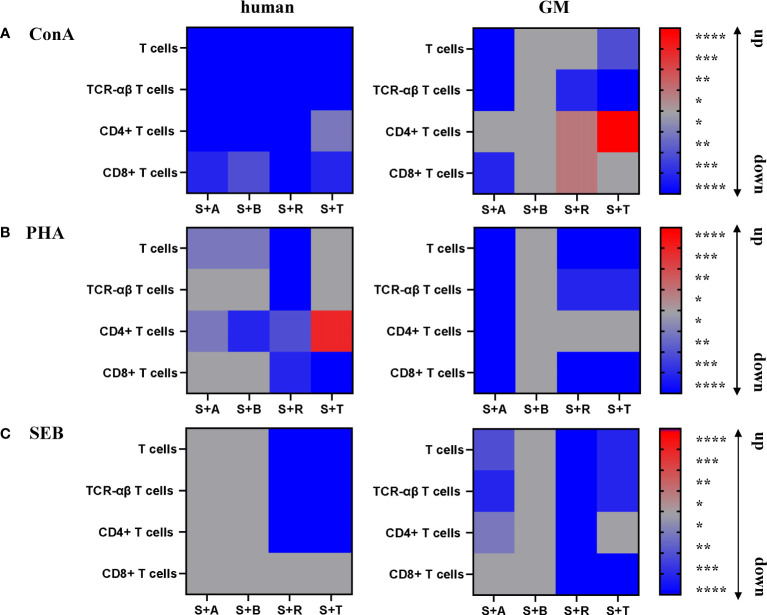
Summary of the stimulatory and inhibitory effects on human and GM PBMC subsets. Proliferative responses of stimulated T cells and subsets of humans and GMs are shown after 5 days of *in-vitro* cultivation with ConA **(A)**, PHA **(B)**, and SEB **(C)** in combination with the four different immunomodulating compounds abatacept (A), belatacept (B), rapamycin (R), and tofacitinib (T). The heatmaps indicate downregulation (blue) or upregulation (red) of proliferative response according to calculated statistical significance shown in **Figures 2**–**5** (*p ≤ 0.1, **p ≤ 0.05, ***p ≤ 0.01, ****p ≤ 0.001).

Abatacept and belatacept, two agents that are closely related and specific to humans, were selected. These compounds inhibit the second T-cell signal by blocking the binding between CD80/86 and CD28. Both compounds consist of a CTLA-4 part, whose extracellular domain is fused to a modified Fc part of human IgG, resulting in a soluble CTLA-4-Ig fusion protein (27–29, 68). To cover two other crucial steps of the T-cell response and activation, we further investigated the master regulator mTOR inhibitor rapamycin and the JAK inhibitor tofacitinib (31, 32, 34, 35). This diversity of modes of action allowed us to address several questions in our study. Our results highlight the importance of being mindful when selecting a stimulus to evaluate the efficacy of a suppressor drug *in vitro* in different species. A suppressor may work very well with one stimulus, but the combination with another stimulus could lead to contradictory conclusions. The results described above are summarized in **Figure 7** as heatmaps for a better overview. Blue shades indicate a downregulation, while red shades indicate an upregulation of proliferation within the different settings.

First, the different stimuli allowed us to study their effects on immunomodulatory compounds. ConA and PHA activate T cells through crosslinking cell-surface receptors and do not correspond to the classical scheme of T-cell activation by TCR and CD28 (69–73). Therefore, we could investigate whether drugs targeting specifically the second T-cell activation signal also have an effect under mitogenic stimulation. This allows for a broader assay capacity rather than being dependent on antigen-specific immune reactivations in the first place. In general, all four compounds showed reducing effects on ConA-stimulated human T-cell subsets. In GM samples, more inhibitory effects were observed in PHA-stimulated cells (**Figures 7A, B**). Here, aside from the treatment with rapamycin and tofacitinib, interestingly, abatacept also showed a reduction of proliferation. Therefore, it is worthwhile to address the question of why abatacept and belatacept show an effect after stimulation with those T-cell mitogens *in vitro*. A possible explanation can be targeting CD80/CD86 expressed by T cells. A previous study showed that human effector memory T cells can express CD86. The authors observed that those cells showed enhanced proliferation and IFN-γ production after PBMCs were stimulated with anti-CD3 mAb and IL-2. Adding neutralizing anti-CD86 mAbs prohibited the effects (74). In another study, the authors found PBMCs expressing CD80 and CD86 *ex vivo*. Through further investigation, they confirmed that CD80 expressed on Tregs was shown to work as a ligand for CTLA-4 (75). One could speculate that through abatacept or belatacept, Tregs are activated, leading to suppression of mitogen activation (76). One big difference between humans and GMs was the promoting effect of tofacitinib on the proliferation of porcine TCR-αβ T cells, particularly the CD4^+^ T-cell subpopulation after stimulation with ConA. It has been reported that Th_2_ and regulatory T-cell lineages are not affected by tofacitinib, while another group reported an upregulation of the frequency of regulatory T cells (T_regs_) after treatment with tofacitinib; hence, it can be speculated that those cells are accountable for the upregulation of CD4^+^ T cells in GMs (77, 78). We will address this issue in future studies by including markers specific for Th_2_ cells and T_regs_ like key transcription factors GATA3 and Foxp3.

Second, superantigens like SEB were reported to show potential to interact directly with CD28 and CD80/86 or strengthen the interaction between these receptors (21, 79). So, potentially, an inhibitory effect of abatacept and belatacept can be expected after SEB stimulation, offering a target for CTLA-4 through CD80/CD86. While the CTLA-4 structure of abatacept is based on a conserved structure, belatacept contains two amino acid substitutions resulting in the replacement of leucine by glutamic acid (position 104) and alanine by tyrosine (position 29) (68). These substitutions are responsible for the increased effect in humans and the stronger blocking of the CD80/86–CD28 interaction (68, 80, 81). This effect could not be confirmed by our data, at least for humans. However, those interactions could also reduce the effect of CD80/CD86 inhibitors if the target is already occupied by the stimuli. Still, a reduction was observed after abatacept treatment in minipigs. Nonetheless, abatacept, regardless of the used stimuli, showed inhibition efficiency on GM PBMCs in our study. Rapamycin showed a comparable suppressive effect on SEB-stimulated PBMCs within total T cells, TCR-αβ T cells, and the CD4^+^ T-cell subpopulation in both species as well as a suppressive effect on CD8^+^ T cells within GMs (**Figure 7C**). In summary, the humanized antibody abatacept showed a promising efficacy on GM PBMCs in combination with the different stimuli, sometimes even achieving a higher reduction compared with human PBMCs. In addition, promising and comparable results between the two species were visible for rapamycin in combination with PHA stimulation.

Additional analyses in the context of an antigen-specific *in-vitro* recall immune response will provide a deeper insight into the effects of suppressors on different subsets of immune cells under conditions that may be closer to the *in-vivo* situation (82–85). This likely complicates the comparison of different species as analogous antigen-specific immune responses must be involved, which may be based on different pathogens or vaccine antigens. We performed the first preliminary test on porcine PBMCs restimulated with baculovirus-expressed PCV2-ORF2 protein causing a recall response after vaccination. Although the frequencies of antigen-specific proliferating cells were lower in comparison to mitogens, inhibitory effects were very well observed in this setting. Likewise, to the polyclonal stimulation, rapamycin led to the highest reduction in proliferation of porcine T cells. In further studies, the effects of immunosuppressive drugs must also be considered concerning different pathogens used in recall assays; for example, tofacitinib has been shown to have a stronger effect on an antiviral than on an antimicrobial immunity *in vivo* in humans (86). A potential vaccine candidate to compare and investigate the compounds in an antigen-specific setting will be influenza A (H1N1), where a genome analysis found gene segments of the swine influenza A (H1N2) in humans (87). Furthermore, T cells of pigs vaccinated with the PHH-1V COVID-19 vaccine candidate showed a promising IFN-γ profile after *in-vitro* restimulation underlining the potential to elaborate more on an antigen-specific restimulation setting (88).

In our experiments, we tested the proliferative capacity of different T-cell subsets, but further assays to study cytokine production will certainly be useful to provide an overall insight. As frozen PBMCs as well as lymphocytes isolated from organs showed potent cytokine production also after thawing in preliminary tests, these assays can be performed in follow-up studies with our minipig samples. In addition to these studies to confirm the effects of a drug, the use of NGS in *in-vitro* studies will deliver important information on the effects within different species by providing information on possibly involved activation pathways.

In conclusion, PBMCs from GM show high potential as an additional alternative to human PBMCs for *in-vitro* testing of immunomodulatory drug candidates prior to the start of *in-vivo*, non-clinical safety studies. These *in-vivo* studies could be challenging, especially for more complex immunomodulatory compounds such as humanized antibodies. However, with genetically modified minipigs, e.g., transgenic for human IgGs (18), these future *in-vivo* studies could give reliable information and might help to reduce and replace non-clinical safety studies in non-human primates.

## Data availability statement

The raw data supporting the conclusions of this article will be made available by the authors, without undue reservation.

## Ethics statement

The studies involving humans were approved by Red Cross Austria. Usage is regulated through “Richtlinie zur Verwendung und Weitergabe von Materialien menschlichen Ursprungs für andere Zwecke als für die Transfusion oder Transplantation beziehungsweise zur Herstellung von Humanarzneimitteln” and internally through the Ethics Commission of Red Cross Austria. The studies were conducted in accordance with the local legislation and institutional requirements. The human samples used in this study were acquired primarily from isolated PBMCs as part of your previous study for which ethical approval was obtained. Written informed consent for participation was not required from the participants or the participants’ legal guardians/next of kin in accordance with the national legislation and institutional requirements. The animal study was approved by the Advisory Committee for Animal Experiments of the University of Veterinary Medicine Vienna (§12 Animal Experiments Act - TVG) and the Austrian Federal Ministry of Education, Science and Research (reference BMBWF-68.205/0198-V/3b/2019). The study was conducted in accordance with the local legislation and institutional requirements.

## Author contributions

CPSP: Conceptualization, Data curation, Formal analysis, Investigation, Methodology, Project administration, Visualization, Writing – original draft. EL: Validation, Writing – review & editing. MS: Methodology, Validation, Writing – review & editing. MD: Data curation, Formal analysis, Writing – review & editing. SJ: Resources, Writing – review & editing. MWS: Conceptualization, Resources, Writing – review & editing. KHM: Conceptualization, Data curation, Formal analysis, Methodology, Validation, Writing – review & editing. AS: Conceptualization, Funding acquisition, Resources, Supervision, Writing – review & editing.
